# Initial diagnosis of Wegener’s granulomatosis mimicking severe ulcerative colitis: a case report

**DOI:** 10.1186/1752-1947-7-141

**Published:** 2013-05-29

**Authors:** Sonja Timmermann, Alberto Perez Bouza, Karsten Junge, Ulf P Neumann, Marcel Binnebösel

**Affiliations:** 1 Department of General, Visceral and Transplantation Surgery, University Hospital of the RWTH Aachen, Aachen, Germany; 2 Institute of Pathology, University Hospital Bonn, Bonn, Germany

## Abstract

**Introduction:**

We describe the case of a woman with an unusual presentation of Wegener’s granulomatosis.

**Case presentation:**

A 20-year old Caucasian woman presented with the principal feature of a pancolonic, superficial microulceration mimicking severe ulcerative colitis. Our patient was refractory to therapy and had persisting signs of septic shock as well as being at risk of perforation, so we performed a subtotal colectomy and a cholecystectomy due to the incipient necrosis of her gallbladder. Histologic analysis of her colon showed multiple superficial microulcera of the mucosa, lamina propria mucosae and, to a lesser extent, the lamina submucosa. The medium-sized arteries and arterioles of her entire colon, appendix and gallbladder showed acute vasculitic changes with fibrinoid necrosis of the walls and diffuse infiltration with neutrophil granulocytes, accompanied by a strong perivascular histiocyte-rich and partially granulomatous reaction. These findings strongly suggested an autoimmune multisystem disease like Wegener’s granulomatosis or microscopic polyangiitis. A diagnosis of Wegener’s granulomatosis was confirmed by the results of serologic antibody tests: her cytoplasmic antineutrophil cytoplasmic antibody titer was considerably elevated at 1:2560 specific for subclass proteinase 3 (>200kU/L). After the histopathological diagnosis and serological tests, immunosuppression with high doses of corticosteroids and plasmapheresis was started.

**Conclusion:**

In critically ill patients with severe, therapy-refractory ulcerative colitis, Wegener´s granulomatosis should be considered and serologic antibody testing should be performed.

## Introduction

Wegener’s granulomatosis is an antineutrophil cytoplasmic antibody (ANCA)-associated vasculitis. This rare autoimmune disease is characterized by a necrotizing granulomatous inflammation of small- to medium-sized vessels and commonly affects both the upper and lower respiratory tract as well as the kidneys. It very seldom involves gastrointestinal organs.

We present a case of Wegener’s granulomatosis as an accidental finding in a woman with symptoms of septic shock and a pancolonic, superficial microulceration of the mucosa mimicking severe ulcerative colitis.

## Case presentation

A 20-year old Caucasian woman in septic shock with multiorgan dysfunction was transferred to our intensive care unit. Her medical history was remarkable for allergic asthma and Basedow’s disease. She had previously undergone a left-sided hemithyroidectomy and right-sided subtotal resection.

About four weeks before admission to the transferring hospital, our patient had been treated with cefuroxime due to a retroareolar inflammation two years after a right-sided breast piercing. Because of the sustained fever and diarrhea, we substituted cefuroxime with metronidazole, suspecting an antibiotic-associated process. Metronidazole was then switched to vancomycin, with the assumption that our patient had pseudomembranous colitis. A colonoscopy showed inflammation and multiple small ulcerations of her entire colon, with the greatest extent in her ileum, cecum and sigma. However, neither pathogen germs nor *Clostridium difficile* toxin could be detected in stool samples and her blood and urine specimens were also sterile. A wound swab of her increasingly necrotic right breast showed *Staphylococcus aureus*, *Actinomyces turicensis* and *Peptostreptococcus* species. Consequently, the progressively damaged tissue was explored and extensively excised to exclude an abscess. Because of the considerable aggravation of her general condition, the antibiotic treatment was again diversified to a three-fold treatment with imipenem and cilastatin, moxifloxacin, and fluconazole. Owing to her hemodynamic and respiratory insufficiency, our patient was transferred to our intensive care unit.

**Figure 1:**
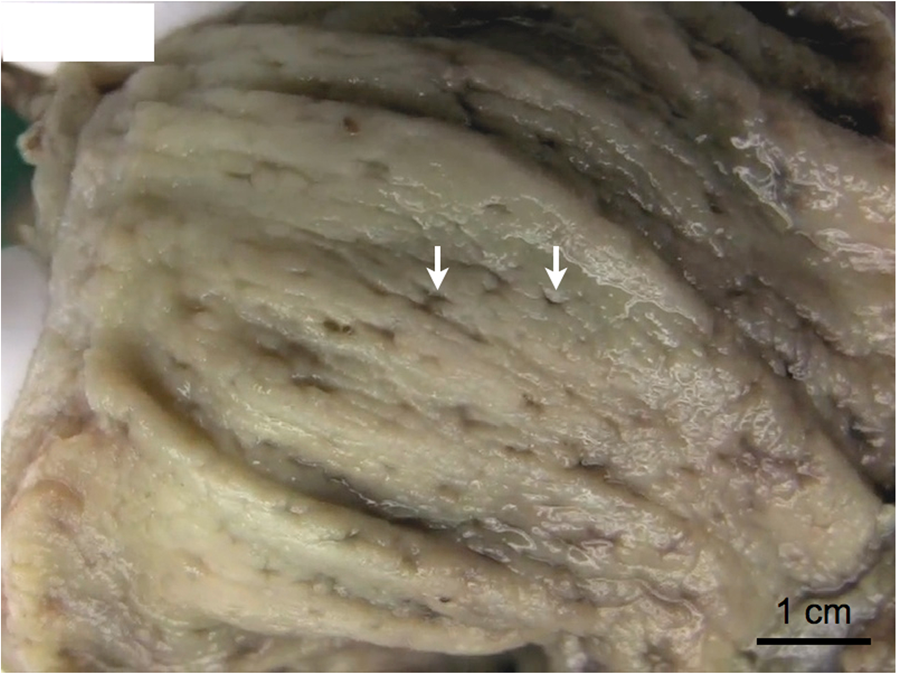
**Macroscopic aspect of the colonic mucosa.** Multiple small ulcerations of a few millimeter diameter were seen dispersed over the entire mucosa of the colon (arrows).

During admission to our ward, ventilation was conducted with 100% oxygen, and our patient needed high catecholamine doses. She was also anuric, with a creatinine level of 5.0mg/dL (reference range 0.7 to 1.2mg/dL) and elevated liver parameters, with total bilirubin 2.9mg/dL (reference range 0.2 to 1.0mg/dL), aspartate transaminase 2572U/L (reference range 10 to 50U/L) and alanine transaminase 608U/L (reference range 10 to 50U/L). She had leukocytosis, with a white blood cell count of 27.0G/L (reference range 4.3 to 10.0G/L). Her C-reactive protein level was >230mg/L (reference range <5mg/dL) and procalcitonin level was 9.3μg/L (reference range 0.1 to 0.5μg/L). An immediate colonoscopy showed multiple ulcerations of the colonic mucosa (Figure 1).

Because our patient was therapy-refractory and had persisting signs of septic shock and a risk of perforation, a subtotal colectomy was indicated. Just before the beginning of the abdominal surgery, her pulmonary gas exchange worsened. When examined by bronchoscopy, there was no evidence of an obstruction; however, the mucosa of her bronchi was highly inflamed and vulnerable. We observed bleeding originating from her upper airway. The ventilatory conditions were instantly ameliorated by a laparotomy - equivalent to the release of intra-abdominal compartment syndrome. Because of the incipient necrosis of her gall bladder, we performed a subtotal colectomy and a cholecystectomy. During the surgery, 20cm of her rectum were left and blindly closed according to Hartmann’s approach, with an ileostomy and a laparostomy.

Postoperatively, we initiated a calculated therapy with meropenem and caspofungin as well as vancomycin to cover a possible translocation of *C. difficile* or its toxins. Furthermore, continuous veno-venous hemofiltration was started.

Permanent stabilization of our patient’s organ functions could not be achieved. Hemodynamic, pulmonary and renal failure still persisted and her liver enzyme levels increased massively (aspartate transaminase 8848U/L, alanine transaminase 1039U/L, total bilirubin 9.4mg/dL), correlating with ischemic necrosis in liver segments six and seven detected by ultrasonic testing. Moreover, our patient showed recurrent ventricular and supraventricular tachycardia culminating in a short-term asystole. Echocardiography did not reveal any pathological changes. All blood, tracheal secretion and abdominal swab samples stayed free of pathological germs. A sudden rise in lactate necessitated a second-look operation, during which we found no evidence of mesenteric ischemia.

**Figure 2:**
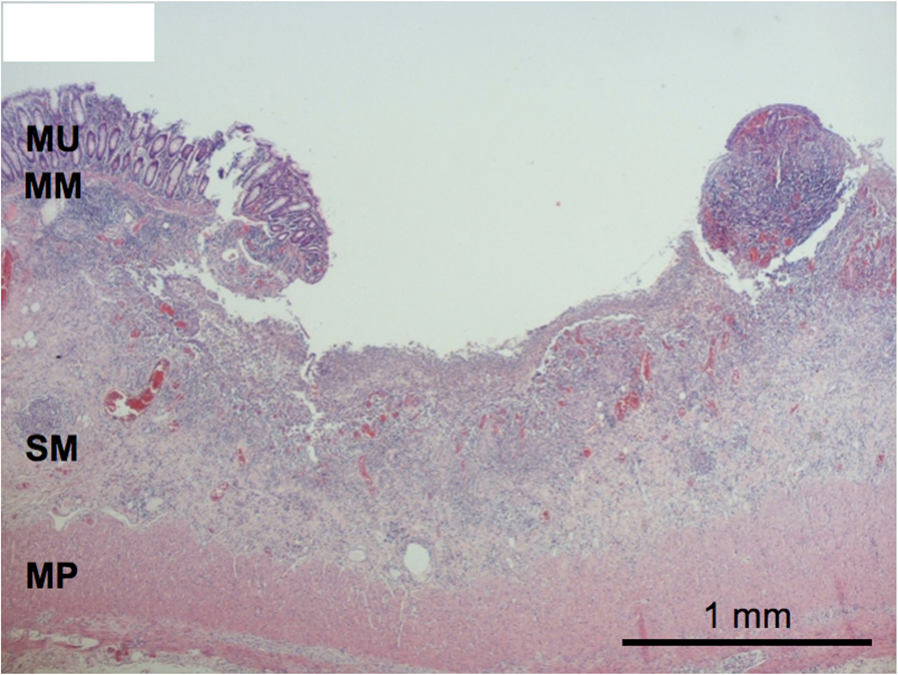
**Histological aspect of one ulcer of the colonic mucosa.** Ulceration is restricted to the mucosa and partially to the submucosa. No alteration of the architecture of the colonic crypts or granuloma formation was found. MM: muscularis mucosa; MP: muscularis propria; MU: mucosa, SM: submucosa.

**Figure 3:**
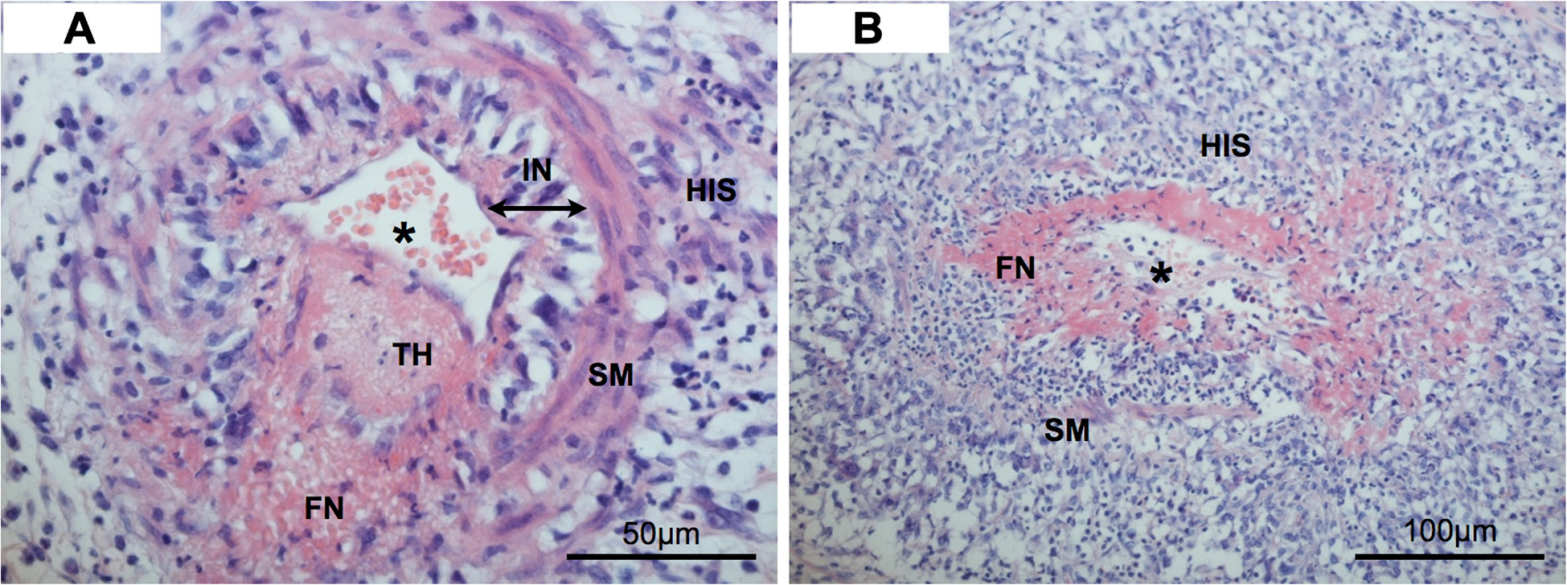
**A and B: Microscopical aspect of two arterioles with vasculitic changes.** (**A**) Vessels are surrounded by dense histiocytic infiltrates forming, in many cases, granulomas. Many vessels also show platelet-rich thrombi associated to inflammatory changes of the wall. (**B**) The vascular wall shows large areas of fibrinoid necrosis with neutrophil granulocytes that were nearly complete destroyed. * lumen of the vessel; FN: fibrinoid necrosis; HIS: histiocytic infiltration; IN: intima; SM: smooth muscle; TH: thrombus.

Histologic examination of her colon showed multiple superficial areas of microulceration of the mucosa, lamina propria mucosae and, to a lesser extent, the lamina submucosa (Figure 2). Medium-sized arteries and arterioles of her entire colon, appendix and gallbladder showed acute vasculitic changes with fibrinoid necrosis of the walls and diffuse infiltration with neutrophil granulocytes, accompanied by a strong perivascular histiocyte-rich and partially granulomatous reaction (Figure 3A,B). Many arterioles also had intraluminal platelet-rich thrombi (Figure 3A), others were complete obliterated by inflammatory cells. The affected vessels were localized in the submucosal layer of her bowel and in her gall bladder. These findings strongly suggested an autoimmune multisystem disease like Wegener’s granulomatosis or microscopic polyangiitis. A diagnosis of Wegener’s granulomatosis was confirmed by the results of the serologic antibody tests: her c-ANCA titer was considerably elevated at 1:2560 specific for subclass proteinase 3 (PR3) (>200kU/L). After the histopathological diagnosis and the serological tests, immunosuppression with high doses of corticosteroids and plasmapheresis were started.

Continuing a dosage of 100mg prednisolone daily and plasmapheresis twice a day for almost a week, we gradually achieved a durable stabilization of our patient’s circulation and lung function, a constant downsizing of the ischemic area in her right liver lobe and a cumulative resumption of urine production. After tapering catecholamines and eliminating about 10L of extravascular fluids, it was possible to close the laparostomy and extubate our patient. Her gas exchange was borderline and she required highly intensive airway treatment and intermittent application of continuously positive airway pressure.

A couple of days later, she developed an acute abdomen and we measured a leap in leukocytes up to 75.0G/L. A computed tomography scan showed multiple hypodense areas in her liver appearing as partial necrosis and her spleen failed to show any contrast at all.

A splenectomy was performed due to multiple septic infarctions, although several samples taken from biopsies of the hepatic lesions were sterile.

Postoperatively, our patient could be extubated without difficulty, her leukocyte level fell and after a perioperative deterioration of kidney function, her creatinine and urea levels stayed within an acceptable range because of reparatory polyuria. She did not show any neurological deficits, slowly regained her strength and could be transferred to a standard ward only five days post-splenectomy.

Plasmapheresis was continued three times a week and prednisolone was gradually reduced to 50mg per day. After completion of wound healing, cyclophosphamide treatment could be initiated.

Retrospectively, we discovered some further, interesting aspects about our patient’s medical history: when she was diagnosed with Basedow’s disease two years before, our patient had positive titers for thyroid peroxidase antibodies (380U/mL; normal range <35U/mL), microsomal antibodies (1:6400; normal range <1:100) and thyroid-stimulating hormone receptor antibodies (29.5U/L; normal range <1U/L). Her parents reported that she had complained about painful knees after exercising and gingival problems for several weeks before exacerbation of the Wegener’s disease.

## Discussion

Primary systemic vasculitides are a rare phenomenon. Because of the variable and also mostly nonspecific symptoms and clinical findings, the correct diagnosis can be delayed or missed. The classification system of all the different vasculitic diseases is a complex matter and currently still subject to change [1]. Fulfillment of certain histological features primarily leads to the diagnosis, which can be confirmed by detection of certain autoantibodies. In this context, the only three disorders frequently associated with ANCAs concern necrotizing small vessel vasculitides such as Wegener’s granulomatosis, microscopic polyangiitis and Churg-Strauß syndrome, including their localized forms [2]. Further differentiation of these antibodies regarding their staining pattern and their target structures makes them important tools for deducing the final diagnosis even though they do not count towards the diagnostic criteria [3]. Excluding atypical ANCA, perinuclear ANCA typically found in microscopic polyangiitis mainly target myeloperoxidase; by contrast, cytoplasmic ANCA against PR3 can be found in about 80% of patients with Wegener’s granulomatosis depending on disease activity [4,5].

Looking at Table 1, it is obvious that determination of an existing ANCA-associated disorder affecting small-to-medium-sized vessels mainly depends on histological and clinical characteristics [6]. In our case, microscopic polyangiitis was ruled out by the ubiquitously present granuloma. The diagnosis of a Churg-Strauß syndrome did not fit because our patient did not have peripheral eosinophilia in the differential blood count. However, all the findings perfectly matched Wegener’s granulomatosis.

**Table 1:** **Positivity of antineutrophil cytoplasmic antibody subclasses in generalized Wegener’s granulomatosis, microscopic polyangiitis and Churg-Strauß syndrome** [[6]]

	**Wegener’s granulomatosis**	**Microscopic polyangiitis**	**Churg-Strauß syndrome**
Proteinase 3 +	70% to 80%	30%	30%
Myeloperoxidase +	10%	60%	30%

Even though Wegener’s granulomatosis can affect almost any organ, it classically causes a triad of hemoptysis, dyspnea and hematuria. Further symptoms may include fatigue, weight loss and elevated temperatures; sinusitis and skin sores as well as dysfunction of eyes or ears can also be related to vasculitic changes. Sometimes cardiac and cerebral vessels are affected. Retrospectively, in our patient the joint aches and periodontitis-like symptoms could be interpreted as first signs of the manifesting disease [7].

Gastrointestinal involvement only rarely emerges clinically although Walton *et al*. found evidence of bowel disease in almost a quarter of patients with Wegener’s granulomatosis at autopsy [8]. There are several published studies that do not mention typical symptoms such as abdominal pain, hematochezia or diarrhea [9-11]. However, since 1982, more and more - often severe - cases have been described, always occurring during early stages of the disease. In most reports, Wegener’s granulomatosis was a pre-known condition in patients developing abdominal grievances; therefore, in some cases it remains unclear if the gastrointestinal involvement resulted from the underlying vasculitic disorder or was an undesirable effect of immunosuppressant treatment [12-15]. According to the chronological sequence of events in our patient, intestinal symptoms were definitely caused by the disease itself.

As many authors before have been, we were also misguided by possible and more common differential diagnoses like inflammatory and infectious bowel diseases [13,16-18]. In contrast to previous cases, our patient had an acute onset of severe colitis after weeks of antibiotic medication without a history of systemic disorders that would have made us think of an autoimmune etiology [19,20].

Concerning the treatment of abdominal Wegener’s granulomatosis, applying or intensifying immunosuppressant therapy can be sufficient [16,17,20]. Yet fulminant courses due to perforation or widespread ischemia regularly require surgical intervention [12,14,21,22], four cases - including ours - even necessitated subtotal colectomy [13,15]. Despite the large resected specimen, it remains a challenge to find histological signs of vasculitis or even typical granulomas. According to Storesund *et al*., only three out of seven biopsies and preparations showed necrotizing vasculitis, granulomas could only be detected once [15]. In a study of patients with different kinds of vasculitic disorders, three out of six colon biopsies were falsely negative [23]. Deniz *et al*. demonstrated ileal involvement [21]; we could also produce exemplary histologic sections displaying necrotizing granulomatous vasculitis from our patient’s colon, appendix and gall bladder.

To avoid severe complications, it is essential to find histological changes in the vessels and to interpret these gastrointestinal events as part of a systemic autoimmune disease. Specimens from mucosal biopsies, however, can test negative because of the deep location and distribution of the affected vessels.

The threat to life from this situation is shown not only by our experience with our patient but also by the case of a 46-year-old man who, despite suturing of three ileal perforations, died of septic shock seven days postoperatively because of two additional perforations and multiple ulcers [12].

Symptomatic involvement of the spleen as seen in our patient is another rare finding in Wegener’s granulomatosis. Walton *et al*. found focal necrotizing arteriolitis of the spleen in 42 out of 54 cases at autopsy [8], however most patients never develop any symptoms during their lifetime. In most instances, chronic splenic infarction is incidentally detected via computerized tomography [21,24-28]. In a review, eight out of 18 patients developed symptoms and histologic confirmation of the diagnosis could only be obtained once [28]. Again, we present a condition that is clinically seldom seen with an adequate pathologic correlate.

Another item that should not be neglected in this discussion regards certain staphylococcal antigens triggering ANCA and provoking relapses of the associated disorder [29,30]. Because *Staphylococcus aureus* was detected in an initial swab of our patient’s breast, some bacterial peptides complementary to parts of PR3 might have induced the production of autoantibodies against PR3, leading to the manifestation of Wegener’s granulomatosis. Otherwise the initial abscess might have been a cutaneous lesion caused by vasculitis; even though all sections of the excised tissue were reviewed after establishing the diagnosis, no vasculitic signs could be found.

## Conclusion

The way in which Wegener’s granulomatosis was diagnosed in our patient is unusual. To the best of our knowledge, there has not been a previous presentation of the disease similar to that in our patient: a young patient in septic shock with multiorgan failure involving lungs, kidneys and liver following severe ubiquitous colitis and gangrenous inflammation of the gall bladder - a life-threatening condition requiring subtotal colectomy and cholecystectomy. The histopathological findings made us completely change our therapeutic concept and thus save this patient’s life.

## Consent

Written informed consent was obtained from the patient for publication of this case report and accompanying images. A copy of the written consent is available for review by the Editor-in-Chief of this journal.

## Competing interests

The authors declare that they have no competing interests.

## Authors’ contributions

ST, KJ and MB collected and interpreted the data and were major contributors in writing and revising the manuscript. APB performed the histochemistry and immunohistochemistry and was a major contributor in writing the manuscript. UPN was involved in interpretation of the data and revised the manuscript critically. All authors read and approved the final manuscript.
